# Conditional ablation of RGS2 in myeloid cells does not influence blood pressure and organ damage in angiotensin II‐induced hypertension in mice

**DOI:** 10.14814/phy2.71023

**Published:** 2026-07-19

**Authors:** Pablo Nakagawa, Ko‐Ting Lu, John J. Reho, Mina Ghobrial, Kathren Kaminski, Ana Hantke‐Guixa, Natalia M. Mathieu, Daniel T. Brozoski, Nisita Chaihongsa, Kelsey K. Wackman, Nikhil R. Rajendiran, Zahra Dhoondia, Jeffrey L. Segar, Curt D. Sigmund, Justin L. Grobe

**Affiliations:** ^1^ Department of Physiology Medical College of Wisconsin Milwaukee Wisconsin USA; ^2^ Cardiovascular Center, Medical College of Wisconsin Milwaukee Wisconsin USA; ^3^ Neuroscience Research Center, Medical College of Wisconsin Milwaukee Wisconsin USA; ^4^ Comprehensive Rodent Metabolic Phenotyping Core Medical College of Wisconsin Milwaukee Wisconsin USA; ^5^ Department of Pediatrics Medical College of Wisconsin Milwaukee Wisconsin USA; ^6^ Department of Biomedical Engineering Medical College of Wisconsin Milwaukee Wisconsin USA

**Keywords:** angiotensin, hypertension, inflammation, innate immunity, RGS2

## Abstract

The role of the immune system in the development of hypertension is well‐established. Recent studies revealed the critical role of G‐protein‐coupled receptor signaling, particularly angiotensin II (Ang II) type 1 receptor (AT1R), in immune cell function. Regulator of G‐protein signaling (RGS) proteins are critical players in terminating G‐protein‐coupled receptor signaling, with RGS2 being particularly interesting because it limits signaling downstream of AT1R. Importantly, RGS2 is highly expressed in myeloid immune cells. Global RGS2 deletion results in elevated blood pressure and Ang II‐dependent vascular hypercontractility. While evidence supports a key role for RGS2 in vascular tissue, its role in immune cells remains underexplored. Herein, we hypothesized that genetic ablation of RGS2 in myeloid cells would amplify Ang II signaling, thereby exacerbating the inflammatory response to Ang II. To test this hypothesis, we employed a conditional myeloid‐specific RGS2 knockout mouse model (RGS2^LysM‐KO^) subjected to chronic Ang II treatment. Contrary to our expectations, both Control and RGS2^LysM‐KO^ exhibited comparable changes in blood pressure, cardiac hypertrophy, albuminuria, renal tubular injury markers, vascular dysfunction, and inflammatory profiles in response to Ang II. Altogether, we conclude that myeloid RGS2 does not significantly affect blood pressure nor modulate renal injury or vascular dysfunction effects of Ang II.

## 1 INTRODUCTION

Hypertension is a highly prevalent chronic disease worldwide and a leading modifiable risk factor for cardiovascular morbidity and mortality. Specifically, hypertension substantially increases the risk of coronary artery disease, stroke, heart failure, atrial fibrillation, chronic kidney disease, and vascular dementia, and is a major driver of global disability and premature death. (Fuchs & Whelton, 2020) Population studies applying the current American College of Cardiology/American Heart Association definition for hypertension (which lowered the diagnostic threshold from >140/90 to >130/80 mmHg), estimated a prevalence of hypertension of ~45%–46% in U.S. adults. (Muntner et al., 2018) Consistently, the National Center for Health Statistics reported a similar estimate of ~47.7%. (Fryar et al., 2024) The dramatic prevalence and catastrophic consequences of persistent elevations in blood pressure demand urgent public health attention to understand the mechanisms that drive hypertension and target organ injury. (Colantonio et al., 2018).

In the last two decades, a growing body of experimental and clinical evidence has implicated both the innate and adaptive immune systems in the initiation and maintenance of high blood pressure and in hypertensive target‐organ injury. (Hunter & Harrison, 2026) Early studies showed that T lymphocytes accumulate in the kidney, vasculature, and perivascular adipose tissue during hypertension, and that genetic or experimental perturbation of adaptive immune cell function alters blood pressure responses to classic hypertensive stimuli, including angiotensin II (Ang II) or deoxycorticosterone acetate. (Guzik et al., 2007) More recent studies provided new evidence that innate immune cells, and in particular myeloid antigen‐presenting cells, are required for the full development of hypertension. (Hevia et al., 2018; Wenzel et al., 2011) Mechanistically, using dendritic cell transfer experiments, it has been demonstrated that hypertensive stimuli increase the formation of reactive γ‐ketoaldehyde adducts in myeloid antigen‐presenting cells, driving T cell activation, proinflammatory cytokine production, and hypertension. (Kirabo et al., 2014) This supports a model in which activation of the innate immune system primes and sustains pathogenic adaptive responses that elevate blood pressure and promote renal and vascular dysfunction and damage. (Barbaro et al., 2017; Harrison et al., 2010; Justin Rucker & Crowley, 2017; Kirabo et al., 2014; Rudemiller & Crowley, 2016) Regulator of G‐protein signaling (RGS) protein family members act as molecular “brakes” on heterotrimeric G protein signaling by accelerating Gα subunit GTP hydrolysis and thereby hastening the termination of G protein‐coupled receptor (GPCR)‐initiated signals. (Kehrl & Sinnarajah, 2002; Perschbacher et al., 2018) While many RGS family members terminate Gi‐mediated signaling, RGS2 is additionally a potent negative regulator of Gq‐mediated signaling and has been shown to limit signaling downstream of the angiotensin II type‐1 receptor (AT1R) evidenced by multiple studies. (Grant et al., 2000; Matsuzaki et al., 2011; Semplicini et al., 2006; Zhang et al., 2011) First, it has been demonstrated that RGS2 attenuates Ang II‐evoked Ca^2+^ responses in HEK293T cells stably expressing the AT1R. RGS2 also limits Ang II induced Ca^2+^/PKC signaling in vascular smooth muscle cells. Ang II‐induced profibrotic responses are blunted by RGS2 in cardiac fibroblasts, and RGS2 suppresses Ang II‐stimulated aldosterone production in human adrenocortical H295R cells. Notably, we have demonstrated that placentas from human pregnancies complicated by preeclampsia exhibit reduced RGS2 expression, and that genetic ablation of RGS2 in the fetoplacental unit in mice results in a preeclampsia‐like phenotype. (Perschbacher et al., 2020) Consistent with these findings, mice lacking RGS2 globally develop hypertension with exaggerated and prolonged vasoconstrictor responses in vivo, and global RGS2 deletion also sensitizes animals to Ang II‐dependent increases in blood pressure and vascular/myogenic tone, indicating that loss of RGS2 predisposes to hypertension. (Hercule et al., 2007; Heximer et al., 2003).

Based on the roles of RGS2 as a negative regulator of G‐protein/AT1R signaling and on evidence that myeloid cells including macrophages and dendritic cells express components of the renin‐angiotensin system and influence Ang II‐driven inflammation and organ injury (Ma et al., 2011; Wu et al., 2020; Yamamoto et al., 2011; Yang et al., 2022), we hypothesized that genetic ablation of RGS2 would amplify Ang II signaling in innate immune cells and thereby exacerbate the hypertensive response and Ang II‐induced renal and vascular damage. To test this, we assessed whether conditional loss of RGS2 in myeloid cells exacerbates blood pressure elevation and augments end‐organ injury in a model of hypertension induced by chronic Ang II infusion.

## 2 METHODS

### 2.1 Animal subjects

This study used a conditional mouse model in which the *Rgs2* gene is ablated in myeloid cells (RGS2^LysM‐KO^). The *Rgs2* ^Flox^ mouse model was originally developed by our team at the University of Iowa on the B6SJLF1/J background, and later donated to the Jackson Laboratories after backcrossing to the C57BL/6J background (Stock No. 037619) (Ritter et al., 2022) This model contains loxP sites flanking exons 2–4 of the *Rgs2* gene. In the presence of Cre recombinase, deletion of exons 2–4 results in a nonfunctional *Rgs2* product. To target myeloid cell lineage, *Rgs2* ^Flox^ mice on the B6SJLF1/J background were crossed with LysM‐cre mice (Jackson Laboratories, Stock No. 004781).

Housing conditions were previously described in detail (Sandgren et al., 2018) Mice were provided with a commercially available phytoestrogen‐free diet (Inotiv 2920x) and filtered/chlorinated tap water was available ad libitum during the experimental procedures. All animals were housed at standard room temperature (∼22°C) under a 14:10 light–dark cycle (light onset at 5 am). Surgical and experimental procedures adhered to the National Institutes of Health's “Guide for the Care and Use of Laboratory Animals” and were approved by the Medical College of Wisconsin and Use Committees.

### 2.2 Angiotensin II‐induced hypertension

Animals were assigned to groups based on sex and genotype. Then, both male and female RGS2^LysM‐KO^ mice and RGS2^LysM‐WT^ littermate controls (12–14 weeks old) were randomly assigned to receive vehicle or Ang II infusion. Ang II (#A9525, Sigma Aldrich) was delivered subcutaneously at 1 μg/kg/min via osmotic minipumps (Alzet model 1004). Prior to pump implantation, animals underwent 2 weeks of habituation to tail‐cuff blood pressure measurements. Pumps were prepared under aseptic conditions with either Ang II or vehicle (saline with 0.01 N acetic acid), filled and primed for 4–6 h. Mice were anesthetized with inhaled isoflurane, the dorsal interscapular region was shaved and sterilized, and a ~ 1 cm mid‐dorsal incision (between the ears) was made to place the minipump subcutaneously. Incisions were closed with 6–0 silk sutures, postoperative analgesia was administered, and mice were kept on a warming pad during recovery. Systolic blood pressure was followed by tail‐cuff for 3 weeks. At week 4, animals were euthanized by pentobarbital overdose. Kidneys were exercised, weighed, and snap‐frozen in liquid nitrogen for RNA and protein assays. Additional organs (including adipose tissue, spleen, liver, heart and lungs) were weighed, flash‐frozen, and stored at −80°C.

### 2.3 Systolic blood pressure by tail cuff plethysmography

Systolic blood pressure (SBP) was recorded with a computerized tail‐cuff plethysmography system (Visitech 2000) as previously described. (Nakagawa et al., 2020) Measurements were taken daily between 8:00 am and 12:00 pm in a quiet room. Animals were acclimated to the procedure for 2 weeks before data collection: during training, mice were restrained on the apparatus and exposed to 40 pressure cycles in sessions performed at least five times per week. All training session data were discarded. On measurement days, each mouse was placed on a platform maintained at 37°C and allowed ten acclimation cycles, after which 30 consecutive SBP readings were acquired and averaged to produce that day's value. Weekly SBP values presented in the study are the mean of five daily averages obtained on five separate days within the same week. Previously we demonstrated that this protracted approach to acclimating mice to the tail‐cuff apparatus and averaging within animal across multiple recording days yielded light phase SBP values that were indistinguishable from SBP values determined during the light phase via radiotelemetric implants in freely moving mice. (Sandgren et al., 2018).

### 2.4 Assessment of aortic stiffness by non‐invasive pulse‐wave velocity (PWV)

PWV was measured at 4 weeks post Ang II infusion using a Doppler ultrasound system (Mouse Doppler TM, Indus Instruments, Webster, TX) as previously described. (Fang et al., 2022) Briefly, mice were anesthetized with 1.5%–2.0% isoflurane and prepared for non‐invasive PWV measures. The thoracic and abdominal aortic regions were shaved using hair‐removal cream (Nair, Church & Dwight, Ewing, NY). Then, animals were placed on a temperature/electrocardiogram (ECG) platform in a supine position and paws were taped to the ECG electrodes. One 20 Mhz Doppler probe was placed on the skin at the level of thoracic aorta and another probe on the skin around the abdominal aorta. Transit times of pulse pressure waves were calculated and the distance between the abdominal and descending aorta was measured using a caliper. PWV is calculated by dividing the abdominal‐to‐descending aorta distance and the transit time.

### 2.5 Vascular function studies

Wire myography was employed to evaluate contractile responses to potassium chloride (10–100 mM) and endothelin‐1 (Sigma Aldrich, catalog # E7764; 0.1–100 nM), and relaxation responses to acetylcholine (Sigma Aldrich, catalog # A6625; 0.001–100 μM) and sodium nitroprusside (Sigma Aldrich, catalog # 71778; 0.001–100 μM) in rings prepared from carotid artery as previously described. (Nakagawa et al., 2020) After 4 weeks post Ang II or vehicle infusion, vessels were isolated and dissected with care to preserve endothelial integrity, and perivascular adipose tissue was removed. Arterial segments were maintained in an organ bath filled with warmed, oxygenated Krebs solution (NaCl 118.3 mM, KCl 4.7 mM, CaCl_2_ 2.5 mM, MgSO_4_ 1.2 mM, KH_2_PO_4_ 1.2 mM, NaHCO_3_ 25 mM, glucose 11 mM). Three‐ to four‐millimeter rings were mounted on hooks attached to a force transducer and equilibrated at a resting tension of 0.25 g for at least 30 min. For experiments assessing vasorelaxation, rings were preconstricted with a PGH_2_ analog, U46619 (Cayman Chemical; Catalog# 16450), prior to cumulative addition of vasodilators. Data acquisition was performed using PowerLab and subsequent analysis carried out with Lab Chart software (AD Instruments).

### 2.6 Urine albumin excretion

Spot urine was collected at week 4, and urine albumin concentration was quantified using a commercially available kit (Ethos Biosciences, Albuwell M, product # 1011) following the standard microplate procedure. Samples were run in triplicate, and concentrations (mg/mL) were calculated using the standard curve. Urine albumin was normalized to urine creatinine concentration, which was quantified using a commercially available kit (Stanbio, Product # 0430‐120).

### 2.7 Real‐time quantitative reverse transcription‐polymerase chain reaction

Twenty to thirty milligrams of kidney cortex tissue was homogenized in TRIzol reagent (Thermo Fisher Scientific, Waltham, MA) with a poly‐propylene pellet pestle. The total RNA was isolated using PureLink™ RNA Mini Kit (Invitrogen, catalog # 12183020) and eluted in 30–50 μL of RNase free water. RNA concentration was quantified using a nanodrop 2000 (Thermo Fisher). Five hundred nanograms of total RNA were reverse transcribed using iScript cDNA Synthesis kit (Bio‐Rad, catalog # 1708890). Real‐time PCR was performed utilizing TaqMan probes and Fast Advanced Master mix (catalog # 4444557) from Applied Biosciences following the manufacturer's protocol. Catalog numbers of Taqman probes were Kim1: Mm00506686‐m1, IL‐1β: Mm00434228‐m1, TNF‐α: Mm00443258_m1, NOS2: Mm00440502‐m1, ARG1: Mm00475988‐m1, MRC1: Mm01329362‐m1, Col1a2: Mm00483888_m1, TGFβ1: Mm01178820_m1, 18s: Mm04277571‐s1. Relative gene expression levels were calculated using the Livak & Schmittgen method. (Livak & Schmittgen, 2001).

### 2.8 Flow cytometry analysis

At 4 weeks post‐vehicle or Ang II infusion, mice received heparin (300 U, ip) and were anesthetized with a lethal dose of pentobarbital. Transcardial perfusion with 15 mL of ice‐cold PBS containing 0.1% heparin was performed to clear blood from tissues. Kidneys, spleen, and thoracic aortas were collected to acquire their immune cell profile. Kidneys were enzymatically digested in a solution containing 1 mg/mL collagenase A (#10103578001, Sigma Aldrich), 100 μg/mL DNase (Sigma Aldrich, St. Louis, MO), and 20 mM HEPES in phenol‐free RPMI for 30 min at 37°C. Thoracic aortas were enzymatically digested in a solution containing 1 mg/mL collagenase A (#10103578001, Sigma Aldrich), 1 mg/mL collagenase B (#11088807001, Sigma Aldrich), 100 μg/mL DNase (#10104159001, Sigma Aldrich), 20 mM HEPES, 5% FBS in phenol free RPMI for 30 min at 37°C. Gentle MACS was used to dissociate cells and the single cell suspension was passed through 70‐μm nylon mesh and separated by percoll centrifugation. Cells were blocked with anti‐CD16/32 antibody (#101320, BioLegend) for 5 min, then stained with Alexa Fluor 488‐conjugated anti‐mouse CD45 (#103122, BioLegend), PE‐conjugated anti‐mouse CD11b (#101208, BioLegend), PerCp‐Cy5.5‐conjugated anti‐mouse Gr‐1 (#108428, BioLegend), APC‐conjugated anti‐mouse F4/80 (#123116, BioLegend), APC‐Cy7‐conjugated anti‐mouse CD3 (#560590, BioLegend), Alexa Fluor 700‐conjugated anti‐mouse CD19 (#115528, BioLegend), Brilliant Violet 510‐conjugated anti‐mouse CD4 (#100559, BioLegend), and PE‐Cy7‐conjugated anti‐mouse CD8 antibodies (#552877, BioLegend). To exclude dead cells, LIVE/DEAD® Fixable Aqua Dead Cell Stain Kit (#L34957, Invitrogen) was used. CountBright Absolute Counting Beads (#C36950, Invitrogen) were used to quantify infiltrating cell numbers per whole tissue. Data were acquired using a BD Fortessa flow cytometer, and then neutrophils, macrophages, and T cell phenotypes were analyzed using FlowJo software (version 10.0.0).

### 2.9 Statistical analysis

Throughout, data are presented as mean ± SEM. Sample sizes are reported for each experiment, and individual data points are shown where feasible to allow assessment of data dispersion. When both sexes were reported combined, we indicated each sex using different symbols. The quantitative analyses were performed in a blinded fashion. Data were analyzed using GraphPad Prism software (Version 11.0.0) to perform two‐way ANOVA or mixed‐effects models with repeated measures as appropriate, followed by Sidak's multiple comparison procedures or 2‐tailed unpaired *t*‐test. The statistical analyses were performed for both sexes combined and for sex‐disaggregated data. Outliers were identified by either interquartile range method or the ROUT method with *Q* = 5%. A *p*‐value of less than 0.05 was considered significant.

## 3 RESULTS

### 3.1 Conditional ablation of RGS2 in myeloid cells

**FIGURE 1:**
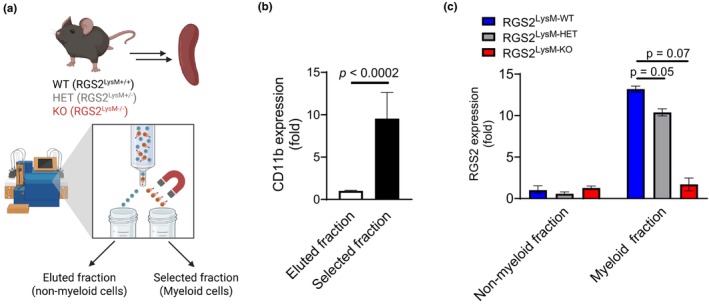
Model Validation of RGS2 Expression in Myeloid Cells. (a) Schematic of magnetic sorting of splenic cells from wildtype (RGS2^LysM‐WT^), heterozygous (RGS2^LysM‐HET^), and homozygous (RGS2^LysM‐KO^) mice. (b) Expression of CD11b in the eluted fraction (non‐myeloid cells) versus the selected fraction (myeloid cells) confirms successful enrichment of myeloid cells in the selected fraction (*p* < 0.0002, unpaired t‐test with Welch's correction). (c) RGS2 expression in eluted (non‐myeloid) and selected (myeloid) splenic fractions from RGS2^LysM‐WT^, RGS2^LysM‐HET^, and RGS2^LysM‐KO^ mice, measured by RT‐qPCR and presented as fold relative to control non‐myeloid cells. Data is presented as mean ± SEM.

The role of RGS2 in innate immunity was studied using a conditional knockout (KO) model, in which mice expressing Cre recombinase under the control of LysM promoter were bred with mice carrying a conditional version of the endogenous *Rgs2* gene. To confirm the selectivity and efficiency of *Rgs2* deletion in myeloid cells, splenic CD11b‐positive cells were isolated using magnetic sorting from wildtype (RGS2^LysM‐WT^), heterozygous (RGS2^LysM‐HET^), and homozygous (RGS2^LysM‐KO^) conditional KO mice. Subsequently, expression of *Itgam* (the gene encoding CD11b) and *Rgs2* were measured by RT‐qPCR in the magnetically selected fraction and the eluted fraction (Figure 1a). As expected, expression of CD11b was significantly elevated in the selected fraction (Figure 1b). In the RGS2^LysM‐WT^ control, the expression of *Rgs2* in the myeloid fraction was higher compared to the non‐myeloid fraction, confirming previous reports that the myeloid cell population expresses abundant RGS2 mRNA. (Yamamoto et al., 2011) Expression of *Rgs2* was blunted in the homozygous RGS2^LysM‐KO^, although it did not reach statistical significance due to small sample size. A partial but significant decrease was observed in the RGS2^LysM‐HET^ (Figure 1c). Consequently, we used the RGS2^LysM‐KO^ conditional KO and compared its phenotype with that of the RGS2^LysM‐WT^ (Control).

### 3.2 Effect of angiotensin II infusion on blood pressure in mice lacking RGS2 in myeloid cells

**FIGURE 2:**
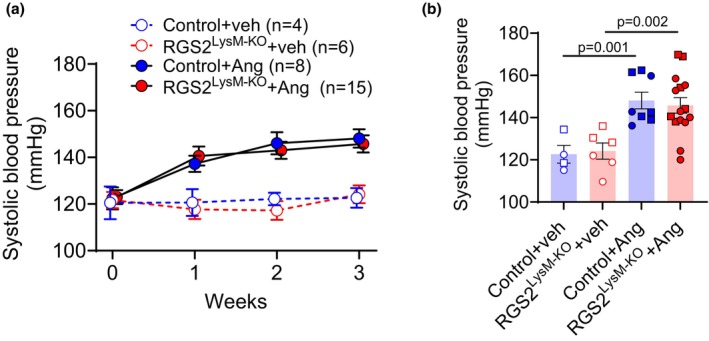
Evaluation of Pressor Response during Ang II Infusion. (a) Systolic blood pressure (SBP) measured by tail cuff plethysmography over 3 weeks of Ang II (1 μg/kg/min) infusion via osmotic minipump. Data are presented as the group mean ± SEM at each time point. (b) Mean SBP presented as a dot‐plot to reveal the distribution of individual animals at Week 3 of Ang II infusion. The error bars reflect the SEM. For statistical analysis, a 2‐way repeated‐measures ANOVA, followed by Sidak's multiple‐comparison post hoc test, was performed. Data from males (squares) and females (circles) were pooled due to the absence of sex differences (Figure S1).

To investigate the role of myeloid cell RGS2 in hypertension, control and KO mice underwent chronic Ang II infusion, and blood pressure was monitored at baseline and for 3 weeks under Ang II treatment. A subset of randomly selected animals was assigned to a control group, which received vehicle instead of Ang II. Ang II effectively increased systolic blood pressure by week 3 in both control and KO with respect to baseline. There was no statistical difference in systolic blood pressures between control and KO mice at baseline or between genotypes during vehicle or Ang II treatments (Figure 2a,b). When comparing systolic blood pressure between males and females (genotypes combined) under Ang II treatment, we observe that males exhibit slightly higher values (Males: 151.1 ± 3.8, *n* = 11 vs. Females: 142.4 ± 3.6, *n* = 12), but this difference was not statistically significant (unpaired *t*‐test; *p* = 0.12). Further, a two‐way ANOVA with sex and genotype as independent variables does not indicate that sex influences the effect of genotype on systolic blood pressure under Ang II treatment (P_sex_ = 0.25, P_genotype_ = 0.89, P_sex×genotype_ = 0.48). Given sex‐specific effects were not observed (Figure S1), we combined both sexes to achieve higher statistical power in subsequent studies. Consistent with the elevated blood pressure, both control and KO mice infused with Ang II exhibited significant hypertrophy evidenced by the elevated heart weight in these animals (Table 1). Liver weight was elevated in KO mice with Ang II infusion compared with vehicle infusion. Under vehicle administration, the KO exhibited a higher spleen mass compared to the control. No differences in body mass, brown adipose tissue, inguinal and perigenital white adipose tissue, kidney, and lung weights were observed between groups, whether sexes were combined or analyzed separately.

**TABLE 1:** Tissue weights after 4 weeks of Ang II treatment.

	Body mass	BAT	iWAT	gWAT	Kidney	Spleen	Liver	Heart	Lung
Control+veh (*n* = 5)	25.50 ± 0.74	2.79 ± 0.42	13.14 ± 2.09	22.38 ± 6.55	6.28 ± 0.21	9.04 ± 4.05	51.28 ± 1.58	4.54 ± 0.10	6.22 ± 0.26
RGS2^LysM‐KO^ + veh (*n* = 7)	26.74 ± 2.16	3.15 ± 0.11	13.83 ± 0.68	21.45 ± 3.47	5.98 ± 0.22	4.47 ± 0.26 #	49.13 ± 1.31	4.65 ± 0.14	5.86 ± 0.30
Control+Ang (*n* = 12)	25.32 ± 1.05	3.06 ± 0.17	11.26 ± 1.73	13.95 ± 1.91	6.35 ± 0.21	5.09 ± 0.53	53.60 ± 1.53	5.50 ± 0.16*	6.33 ± 0.34
RGS2^LysM‐KO^ + Ang (*n* = 14)	24.48 ± 0.85	2.84 ± 0.12	9.96 ± 0.48	15.69 ± 0.91	5.83 ± 0.32	5.11 ± 0.63	53.61 ± 2.00*	5.10 ± 0.12*	6.24 ± 0.24

*Note*: Brown adipose tissue (BAT), inguinal white adipose tissue (iWAT), gonadal white adipose tissue (gWAT), right kidney, spleen, liver, heart, and lungs were isolated from Control and RGS2^LysM‐KO^ mice treated with either vehicle (veh) or angiotensin II (Ang) for 4 weeks. Tissue weights are expressed in grams and normalized to individual body mass. Data are presented as mean ± SEM. Sample sizes for each group are indicated as (*n*). Statistical analysis was conducted using a Two‐Way ANOVA with Uncorrected Fisher's LSD. * *p* < 0.05 versus treatment within genotype; #*p* < 0.05 versus WT within the same treatment.

### 3.3 Effect of angiotensin II infusion on renal injury and immune cell infiltration in mice lacking RGS2 in myeloid cells

**FIGURE 3:**
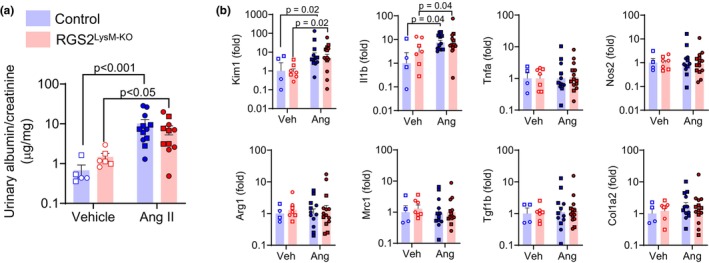
Evaluation of Renal Injury after Ang II infusion. (a) Urinary albumin to creatinine ratio (μg/mg) in Control and RGS2^LysM‐KO^ after 4 weeks of treatment with vehicle (veh) or Ang II. Urine albumin concentration was determined by ELISA, and urine creatinine concentration was determined using a colorimetric assay. Data were log‐transformed and expressed as μg albumin per mg of creatinine. Ang II increased urinary albumin/creatinine in both genotypes, reaching significance in Control+Ang II versus Control+Vehicle (*p* < 0.05, two‐way ANOVA followed by Tukey multiple comparison test). (b) Renal cortical mRNA expression levels of the Kidney Injury Molecule‐1 (Kim1), Interleukin‐1 Beta (Il1b), Tumor Necrosis Factor‐Alpha (Tnfa), Nitric Oxide Synthase 2 (Nos2) (top row), Arginase‐1 (Arg1), Mannose Receptor C‐Type 1 (Mrc1), Transforming Growth Factor Beta 1 (Tgf1b), and Collagen Type 1 Alpha 2 Chain (Col1a2) (bottom row) after 4 weeks of treatment measured by quantitative RT‐PCR and plotted as fold relative to Control+Veh. Data are shown as individual animals (males, squares; females, circles) with bar height reflecting the mean and error bars reflecting SEM. Two‐way ANOVA, followed by Tukey's multiple comparisons test, was performed.

**FIGURE 4:**
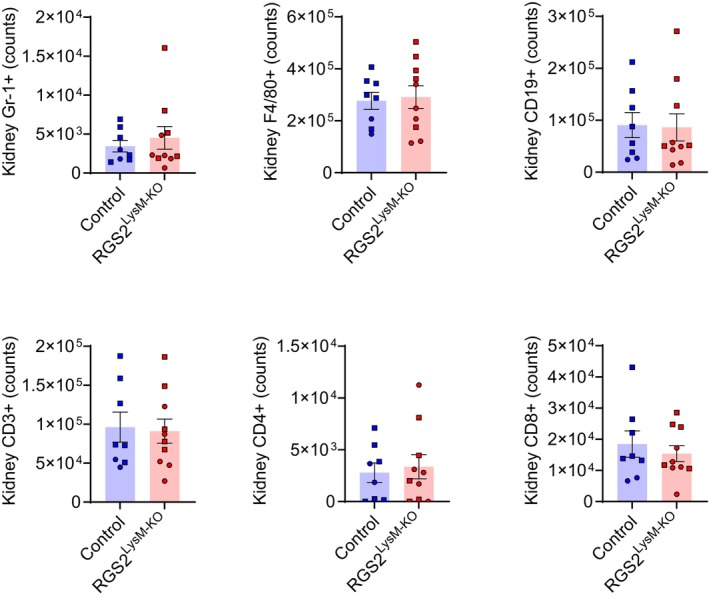
Evaluation of Immune Cell Infiltration in Renal Cortex after Ang II Infusion. Flow cytometric quantification of renal cortex immune cell populations in male Ang II‐treated Control and RGS2^LysM‐KO^ mice. Total counts per tissue are presented for Gr‐1^+^ granulocytes, F4/80^+^ macrophages, CD19^+^ B cells (top row), CD3^+^ total T cells, and CD4^+^ helper or CD8^+^ cytotoxic T cell infiltrates (bottom row). All data are shown as individual male animals with bar height reflecting the mean and error bars reflecting SEM.

It is well accepted that a sustained increase in blood pressure leads to end‐organ damage. Thus, we investigated whether ablation of RGS2 would exacerbate renal damage at 4 weeks of Ang II infusion. When comparing urinary albumin excretion at baseline, it appears that KO exhibits higher albuminuria compared to control. However, these differences are not statistically significant (Figure 3a). Then, we observed that Ang II significantly elevated urinary albumin excretion in both control and KO mice but again, there was no significant difference in albuminuria between control and KO. Then, several markers of renal injury were quantified in the renal cortex by PCR. We observed that expression of *Kim1* and *Il1b*, a surrogate marker of tubular damage and a pro‐inflammatory cytokine, respectively, were elevated in both groups in response to Ang II but no genotype differences were detected (Figure 3b). Several markers of inflammation (*Tnfa, Nos2, Arg1, Mrc1*) and fibrosis (*Tgf1b, Col1a2*) were unaffected by Ang II in either genotype, as expected, since contrary to rats, mice are well known to be more resistant to Ang II renal injury. Because G protein signaling regulates immune cell trafficking and RGS proteins are critical regulators of GPCR signaling by accelerating GTP hydrolysis, we sought to evaluate renal immune cell infiltration using flow cytometry using the gating strategy depicted in Figure S2a. Unexpectedly, control and KO mice exhibited no significant differences in the absolute counts nor percentages of Gr‐1+ granulocyte, F4/80 macrophage, CD19+ B cell, CD3+ total T cell, or CD4+ helper or CD8+ cytotoxic T cell infiltration (Figure 4 and Figure S2b, respectively), suggesting that RGS2 in myeloid cells may not influence the recruitment of immune cells to the kidney during hypertension induced by chronic Ang II infusion.

### 3.4 Effect of angiotensin II infusion on vascular function, stiffness, and immune cell infiltration in mice lacking RGS2 in myeloid cells

**FIGURE 5:**
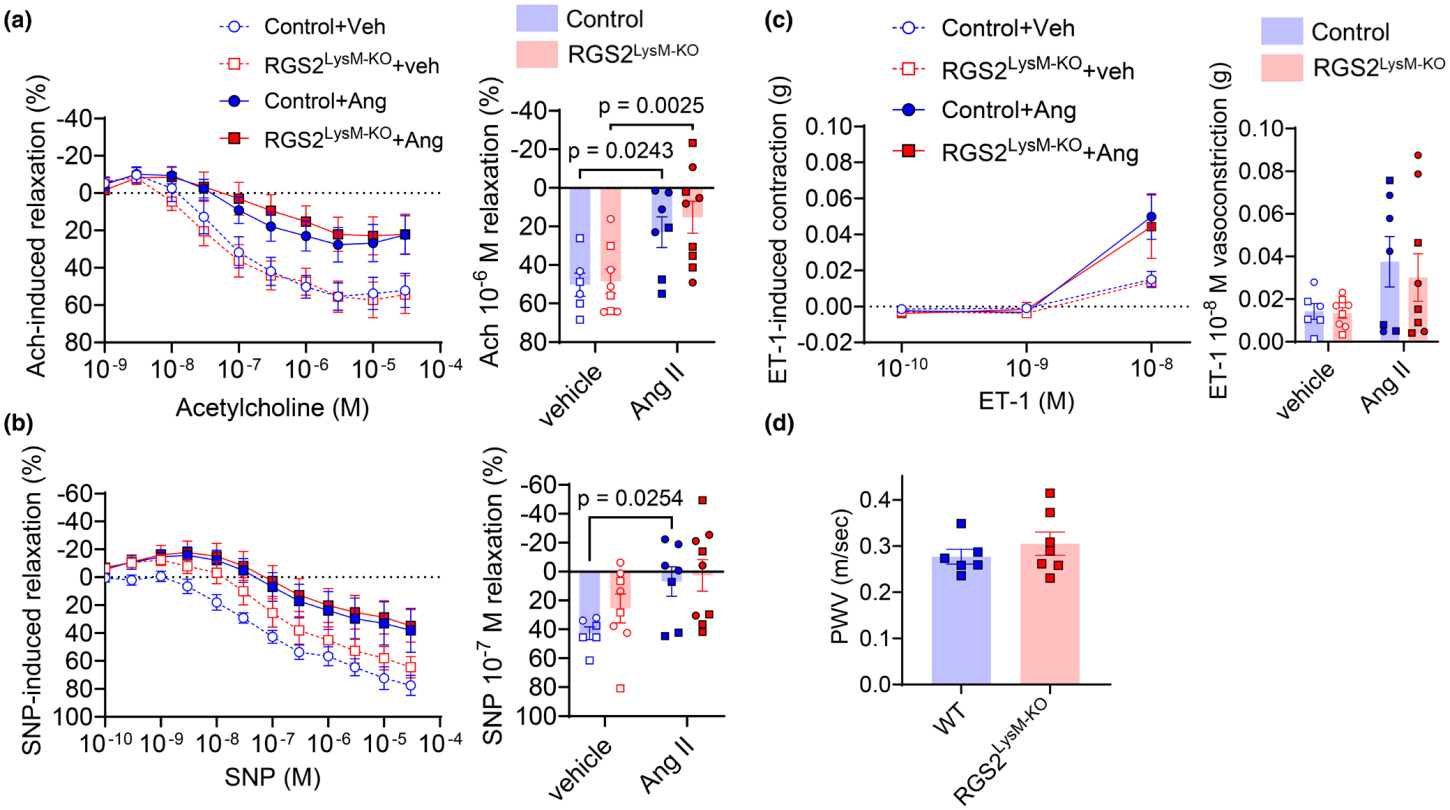
Evaluation of Vascular Function after Ang II Infusion. Vascular function was assessed using wire myography in the carotid arteries of Control and RGS2^LysM‐KO^ treated with vehicle or Ang II (a–c). Dose response curves to (a) Acetylcholine (Ach), (b) Sodium Nitroprusside (SNP), and (c) Endothelin‐1 (ET‐1) (left) and mean percent relaxation or contraction at the specified concentration. (d) Pulse wave velocity (PWV, m/s) was measured to evaluate arterial stiffness in Ang II‐treated Control and RGS2^LysM‐KO^ mice. Bar‐plot data are shown as individual animals (males, squares; females, circles) with bar height reflecting the mean and error bars reflecting SEM and analyzed using a repeated‐measures 2‐way ANOVA with Sidak multiple‐comparisons procedure.

**FIGURE 6:**
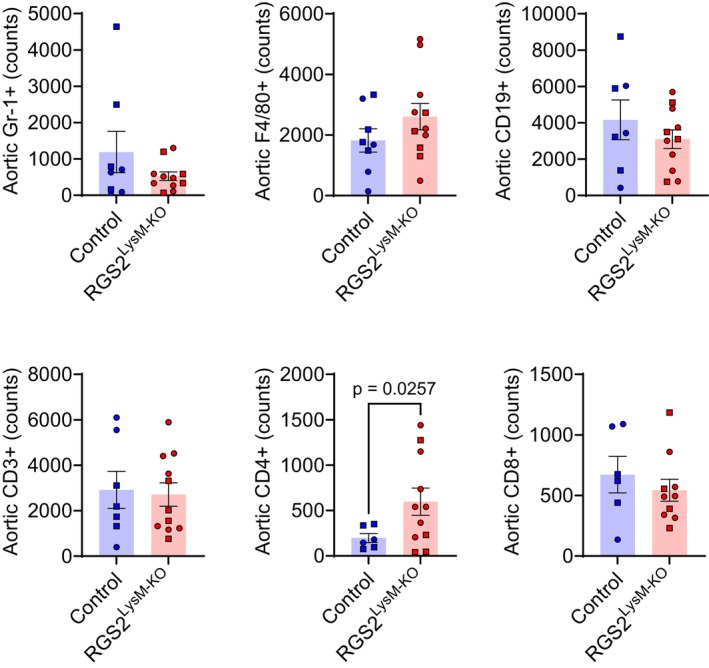
Evaluation of Immune Cell Infiltration in Thoracic Aorta after Ang II Infusion. Flow cytometric quantification of thoracic aorta immune cell populations in male Ang II‐treated Control and RGS2^LysM‐KO^ mice. Counts are presented for Gr‐1^+^ granulocytes, F4/80^+^ macrophages, CD19^+^ B cells (top row), CD3^+^ total T cells, and CD4^+^ helper or CD8^+^ cytotoxic T cell infiltrates (bottom row). All data are shown as individual male animals with bar height reflecting the mean and error bars reflecting SEM and analyzed using. unpaired *t*‐test with Welch's correction.

After 4 weeks of Ang II or vehicle infusion, carotid arteries were isolated from control and KO mice for vascular function studies. First, we evaluated endothelium‐dependent vasorelaxation in response to acetylcholine in preconstricted vessels. In vehicle infused control mice, both control and KO exhibited expected dose‐dependent vasorelaxation and infusion of Ang II for 4 weeks significantly attenuated responses to acetylcholine (Figure 5a). Ablation of RGS2 in myeloid cells did not affect endothelial function relative to control under normal or hypertensive conditions. Second, we evaluated endothelium‐independent vasorelaxation in response to sodium nitroprusside. Mice treated with vehicle exhibited robust vasodilation in response to sodium nitroprusside. Under Ang II treatment, control and KO carotid arteries both similarly exhibited impaired endothelium‐independent vasorelaxation (Figure 5b). Third, we evaluated vasoconstriction in response to endothelin. Under Ang II, both control and KO showed a trend toward an enhanced endothelin‐induced vasoconstriction, but did not reach statistical significance. No differences between control and KO were observed (Figure 5c). Pulse wave velocity, a surrogate indicator of arterial stiffness, was measured in control and KO mice only after Ang II infusion. Again, no differences between control and KO were observed (Figure 5d). Finally, the thoracic aorta was isolated to evaluate immune cell infiltration by flow cytometry analyses and the gating strategy depicted in Figure S3a. Control and KO mice exhibited no differences in CD19+ B cells, CD3+ total T cells, and CD8+ cytotoxic T cell infiltration, but interestingly aortas from the KO exhibited elevated CD4+ T helper cell infiltration and a trend toward increase in macrophages concomitant with a trend toward decrease in granulocytes (Figure 6 and Figure S3b). These immune cells were also evaluated in spleen, and no significant differences were observed between genotypes (Figure S4).

## 4 DISCUSSION

The central objective of this study was to determine the contribution of RGS2 within myeloid cells to the innate immune function, blood pressure, and end‐organ damage responses to chronic Ang II infusion. To accomplish this goal, we developed a conditional myeloid immune cell‐specific RGS2 KO model, which was exposed to chronic infusion of Ang II or vehicle. Because mice are known to be resistant to Ang II‐induced organ injury, we employed a high Ang II dose of 1 μg/kg/min, which typically causes rapid and persistent elevations in blood pressure, glomerular and tubular damage, and significant impairments of vascular function and stiffness.

As expected, at this Ang II dose, both control and myeloid‐specific RGS2‐deficient mice exhibited sustained and significant elevations in blood pressure, albuminuria, and expression of *Kim1* (a marker of tubular damage) and *IL1β* (a pro‐inflammatory cytokine). However, no differences in these effects were observed between control and KO mice. The lack of differences in blood pressure response was not unexpected, as we designed the experiment to prioritize evaluation of renal and vascular end‐organ damage, which are typically not achieved with lower doses of Ang II (e.g., ~490 ng/kg/min) that are often used to unmask differences in blood pressure control. (Ji et al., 2014) That is, we originally hypothesized that the higher dose of Ang II used in this study would induce differential organ damage in response to a similar, maximum effect on blood pressure between genotype groups.

We observed that this Ang II regimen induced prominent renal injury, but this was unaffected by removing RGS2 from innate immune cells. Because previous studies suggest that AT1R signaling in innate immune cells contributes to inflammation and macrophage polarization (Wang et al., 2021; Yang et al., 2022), we hypothesized that RGS2 would confer protective effects, as the termination of AT1R signaling requires RGS2. We also considered studies performed by Dr. Steven Crowley's group, which demonstrated that selective ablation of AT1R in myeloid cells, such as dendritic cells, or bone marrow chimeras lacking AT1R on immune cells, paradoxically aggravates renal injury and blood pressure elevation (Crowley et al., 2010; Lu et al., 2022) Thus, the results of this study, which show that the absence of RGS2 in innate immune cells neither exacerbated nor ameliorated the detrimental effects of Ang II, are surprising. We should note that, in our model, RGS2 ablation occurs in all myeloid cells, not exclusively in AT1R‐expressing myeloid cells. Thus, future studies employing more selective targeting methods, such as combinatorial genomics with Cre and Flp recombinases, could address this limitation. Of note, we determined that this hypertensive model was insufficient to induce macrophage polarization in the kidneys of either group, as no changes in TNF‐α, NOS2, ARG1, and MRC1 were observed. This suggests that at this Ang II dose and time point, there is no profound innate immune modulation. We speculate that inducing a more severe pathological state with a more prominent innate immune response could unmask an inflammatory phenotype in these mice. We also consider T cells could be an important target for RGS2 ablation. Previously, Oliveira‐Dos‐Santos et al. reported that lack of RGS2 results in impaired T cell proliferation and interleukin 2 production ex vivo (Oliveira‐Dos‐Santos et al., 2000) In contrast, a recent preprint report by Raff et al. suggests that T cell‐specific RGS2 KO mice exhibit elevated frequencies of anti‐inflammatory T regulatory cells in the ovary and uterus, thereby enhancing oocyte production (Raff et al., 2024) Future studies would help elucidate whether the immunoregulatory roles of RGS2 are context dependent.

We also consider that the mice used in this study possessed a mixed genetic background, that is, the *Rgs2* ^flox^ dams were on a B6SJLF1/J background, whereas the LysM‐Cre driver line was maintained on a C57BL/6J background. This genetic heterogeneity may have contributed to increased variability in the observed data. Accordingly, it represents a potential confounding factor to consider when interpreting the results and may warrant further control or backcrossing in future studies.

Endothelin 1 is a well‐characterized vasoactive peptide that has been linked to inflammatory processes. (Cunningham et al., 1997; Yanagisawa et al., 1988) In the vasculature, endothelin binds to ETA receptors, resulting in vasoconstriction and high blood pressure. (Kohan et al., 2011) It is proposed that Ang II, oxidative stress, and low‐grade inflammation can enhance ETA signaling cascades. (Böhm & Pernow, 2007; Kostov, 2021; Pernow et al., 2012; Yoshida et al., 1991) Thus, we hypothesized that ablation of RGS2 in myeloid cells would exacerbate ET‐1 effects under hypertensive conditions. Surprisingly, the lack of RGS2 in myeloid cells did not affect endothelium‐dependent or independent vasorelaxation nor exacerbate endothelin 1‐induced vasoconstriction. We observed a mild but significant increase in T helper cell adhesion to the aortic wall, and a trend toward increased macrophage numbers as well. This suggests that RGS2 might modulate immune cell trafficking and adhesion in vessels, but that is insufficient to impair vascular function and stiffness, at least in this mouse model of hypertension with mild inflammation.

Three major limitations of the current study should be noted. First, we did not rule out the possibility that RGS2 in myeloid cells could affect blood pressure responses to a mild hypertensive stimulus, such as lower‐dose Ang II. Future studies using lower doses of Ang II to elicit a milder increase in blood pressure are warranted. Similarly, the effect of RGS2 in these cells to modulate blood pressure and end organ damage in other subtypes of hypertension (e.g., low RAS activity models such as the L‐nitro‐arginine methyl ester or deoxycorticosterone acetate‐salt interventions) should be evaluated, as elevated RAS activity is only observed in roughly 1/6th of all humans with primary hypertension. (Bühler et al., 1973) Second, we acknowledge that blood pressure measurements were obtained using tail‐cuff plethysmography only during the light phase, and that a radiotelemetric approach could presumably detect small differences in blood pressure, circadian variations, and changes in autonomic functions and heart rate control. Third, while several studies have shown the influence of sex and sex hormones in the development of Ang II‐induced hypertension, this study did not reveal overt differences in blood pressure between males and females. (Pollow Jr. et al., 2015; Xue et al., 2005) Thus, the data collected from males and females have been combined to achieve higher statistical power. One of the main differences between our study and previous studies evaluating the influence of sex is that the Ang II dose used in these previous studies was slightly lower (800 ng/kg/min), and, as previously stated, the higher dose used in this study (1000 ng/kg/min) may have achieved a ceiling effect that masked differential blood pressure responses. While we can argue that the tail‐cuff method is not sufficiently sensitive to detect sex differences in blood pressure, it is expected that Ang II would induce a 35.1 ± 5.7 mmHg increase in BP in males versus 7.2 ± 2.0 mmHg in females. (Xue et al., 2005) These differences are high enough to be detected using the tail‐cuff method.

Considering our findings and limitations, we conclude that this study provides evidence that ablation of RGS2 in myeloid cells is insufficient to exacerbate renal and vascular injury under equally high blood pressure levels induced by high doses of Ang II.

## AUTHOR CONTRIBUTIONS

P.N. and J.LG. conceived and designed the study; P.N., K.T.L., J.J.R., A.H.G., D.T.B., N.C., K.K.W. N.R.R., Z.D. performed the experiments and collected the data; P.N., K.T.L., J.J.R., A.H., D.T.B., N.C., K.K.W., N.R,R, and Z.D. analyzed and interpreted the data; P.N., M.G., K.K., and N.M.M, drafted the manuscript; P.N., C.D.S., J.L.S., and J.LG. revised the manuscript for important intellectual content; P.N., C.D.S., J.L.S., and J.LG. acquired funding to conduct the experiments. All authors approved the final version of the manuscript and agree to be accountable for all aspects of the work.

## FUNDING INFORMATION

This study was funded by the National Institutes of Health (HL153101 to PN, HL134850 to JLG; DK133121 to JLS and JLG; T32HL007852 to MG and KK; T32HL134643 to NMM), the American Heart Association (23CDA1048244 to PN and 26POST1550521 to NMM), the Advancing a Healthier Wisconsin Endowment (AHW 9520639 and 5520879 to PN), and the James J. Smith & Catherine Welsch Smith, Mellowes family, and Butenhoff family endowments to MCW.

## CONFLICT OF INTEREST STATEMENT

The authors have no relevant conflicts of interest.

## ETHICS STATEMENT

This manuscript complies with the ethics outlined in the Physiological Reports' Author Guidelines. All procedures adhered to the National Institutes of Health's “Guide for the Care and Use of Laboratory Animals”. The Medical College of Wisconsin and the University of Iowa Animal Care and Use Committees approved this study.

## Supporting information


**Figure S1.** SBP Data from Figure 2 Separated by Sex. (A) Male animals (top) and (B) female animals (bottom). Weekly SBP over 3 weeks of Ang II infusion (left). Week 3 SBP mean ± SEM is represented as a bar graph with individual data points overlaid. No significant differences were observed between sexes within groups; therefore, male and female data were pooled for the analysis presented in Figure 2.
**Figure S2.** Flow Cytometric Gating and Percentages of Renal Leukocyte Subsets. (A) Representative flow cytometry gating strategy for renal cortex leukocyte subpopulations from Ang II‐treated male mice. (B) Percentage of parent gates for Gr‐1^+^ granulocytes, F4/80^+^ macrophages, CD19^+^ B cells (top row), CD3^+^ total T cells, and CD4^+^ helper or CD8^+^ cytotoxic T cell infiltrates (bottom row).
**Figure S3.** Flow Cytometric Gating and Percentages of Thoracic Aorta Leukocyte Subsets. (A) Representative flow cytometry gating strategy for thoracic aorta leukocyte subpopulations from Ang II‐treated male mice. (B) Percentage of parent gates for Gr‐1^+^ granulocytes, F4/80^+^ macrophages, CD19^+^ B cells (top row), CD3^+^ total T cells, and CD4^+^ helper or CD8^+^ cytotoxic T cell infiltrates (bottom row).
**Figure S4.** Flow Cytometric Gating and Percentages of Splenic Leukocyte Subsets. (A) Representative flow cytometry gating strategy for splenic leukocyte subpopulations from Ang II‐treated male mice. (B) Percentage of parent gates for Gr‐1^+^ granulocytes, F4/80^+^ macrophages, CD19^+^ B cells (top row), CD3^+^ total T cells, and CD4^+^ helper or CD8^+^ cytotoxic T cell infiltrates (bottom row).

## Data Availability

All data will be available on reasonable request.
