# Supplementary material for: Conditional ablation of RGS2 in myeloid cells does not influence blood pressure and organ damage in angiotensin II‐induced hypertension in mice
Source: Physiol Rep. 2026 Jul 19;14(14):e71023. doi: 10.14814/phy2.71023 (PMC13382158; doi:10.14814/phy2.71023)
Supplement: Supplementary file 1 — Figure S1. SBP Data from Figure 2 Separated by Sex. (A) Male animals (top) and (B) female animals (bottom). Weekly SBP over 3 weeks of Ang II infusion (left). Week 3 SBP mean ± SEM is represented as a bar graph with individual data points overlaid. No significant differences were observed between sexes within groups; therefore, male and female data were pooled for the analysis presented in Figure 2. Figure S2. Flow Cytometric Gating and Percentages of Renal Leukocyte Subsets. (A) Representative flow cytometry gating strategy for renal cortex leukocyte subpopulations from Ang II‐treated male mice. (B) Percentage of parent gates for Gr‐1+ granulocytes, F4/80+ macrophages, CD19+ B cells (top row), CD3+ total T cells, and CD4+ helper or CD8+ cytotoxic T cell infiltrates (bottom row). Figure S3. Flow Cytometric Gating and Percentages of Thoracic Aorta Leukocyte Subsets. (A) Representative flow cytometry gating strategy for thoracic aorta leukocyte subpopulations from Ang II‐treated male mice. (B) Percentage of parent gates for Gr‐1+ granulocytes, F4/80+ macrophages, CD19+ B cells (top row), CD3+ total T cells, and CD4+ helper or CD8+ cytotoxic T cell infiltrates (bottom row). Figure S4. Flow Cytometric Gating and Percentages of Splenic Leukocyte Subsets. (A) Representative flow cytometry gating strategy for splenic leukocyte subpopulations from Ang II‐treated male mice. (B) Percentage of parent gates for Gr‐1+ granulocytes, F4/80+ macrophages, CD19+ B cells (top row), CD3+ total T cells, and CD4+ helper or CD8+ cytotoxic T cell infiltrates (bottom row). [file PHY2-14-e71023-s001.docx]

**Conditional Ablation of RGS2 in Myeloid Cells Does Not Influence Blood Pressure and Organ Damage in Angiotensin II-Induced Hypertension**

Pablo Nakagawa^1,2,3^, Ko-Ting Lu^1^, John J. Reho^1,6^, Mina Ghobrial^1^, Kathren Kaminski^1^, Ana Hantke-Guixa^1^, Natalia M. Mathieu^1^, Daniel T. Brozoski^1^, Nisita Chaihongsa^1^, Kelsey K. Wackman^1^, Nikhil R. Rajendiran^1^, Zahra Dhoondia^1^, Jeffrey L. Segar^1,4^, Curt D. Sigmund^1,2,3^, Justin L. Grobe^1,2,3,5,6^

^1^Department of Physiology, Medical College of Wisconsin, Milwaukee, WI 53226

^2^Cardiovascular Center, Medical College of Wisconsin, Milwaukee, WI 53226

^3^Neuroscience Research Center, Medical College of Wisconsin, Milwaukee, WI 53226

^4^Department of Pediatrics, Medical College of Wisconsin, Milwaukee, WI 53226

^5^Department of Biomedical Engineering, Medical College of Wisconsin, Milwaukee, WI 53226

^6^Comprehensive Rodent Metabolic Phenotyping Core, Medical College of Wisconsin, Milwaukee, WI 53226

Running Title: Role of Myeloid RGS2 in Hypertension

Correspondence:

Pablo Nakagawa, Ph.D.

Assistant Professor

Department of Physiology

Medical College of Wisconsin

8701 Watertown Plank Road

Milwaukee, WI 53226-0509

Email: [pnakagawa@mcw.edu](mailto:pnakagawa@mcw.edu)

Keywords: RGS2, hypertension, inflammation, innate immunity, angiotensin,


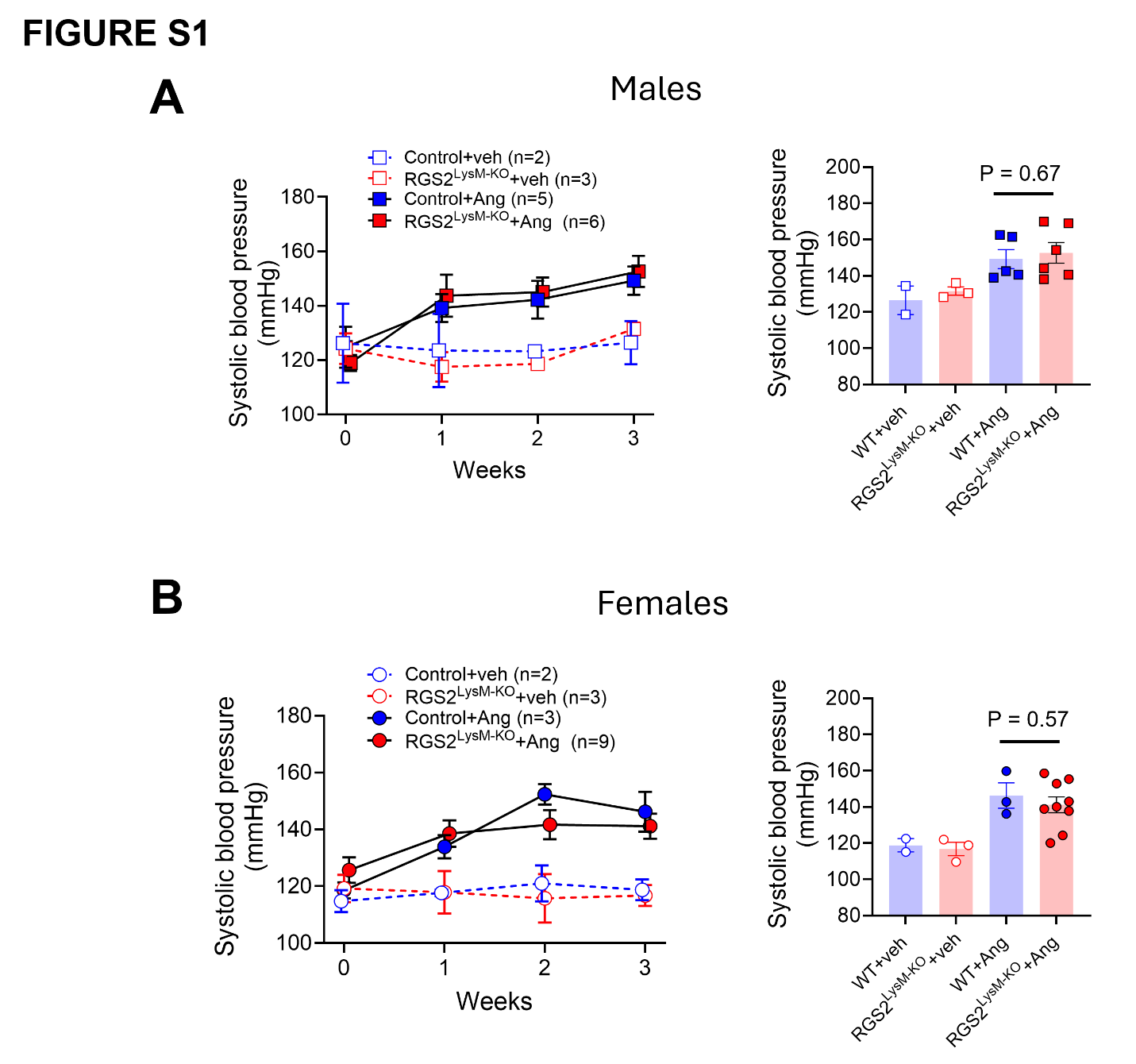


Figure S1: SBP Data from Figure 2 Separated by Sex. A) Male animals (top) and B) female animals (bottom). Weekly SBP over 3 weeks of Ang II infusion (left). Week 3 SBP mean ± SEM is represented as a bar graph with individual data points overlaid. No significant differences were observed between sexes within groups; therefore, male and female data were pooled for the analysis presented in Figure 2.


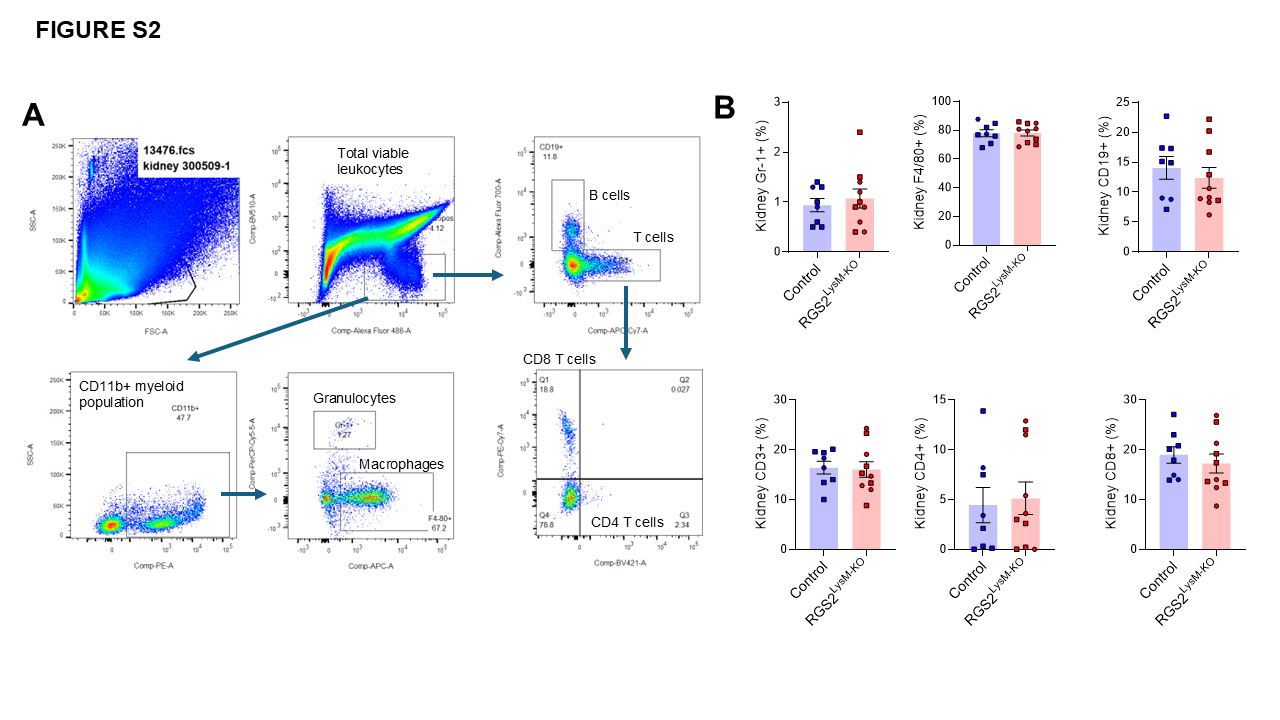


Figure S2: Flow Cytometric Gating and Percentages of Renal Leukocyte Subsets. A) Representative flow cytometry gating strategy for renal cortex leukocyte subpopulations from Ang II-treated male mice. B) Percentage of parent gates for Gr‑1⁺ granulocytes, F4/80⁺ macrophages, CD19⁺ B cells (top row), CD3⁺ total T cells, and CD4⁺ helper or CD8⁺ cytotoxic T cell infiltrates (bottom row).


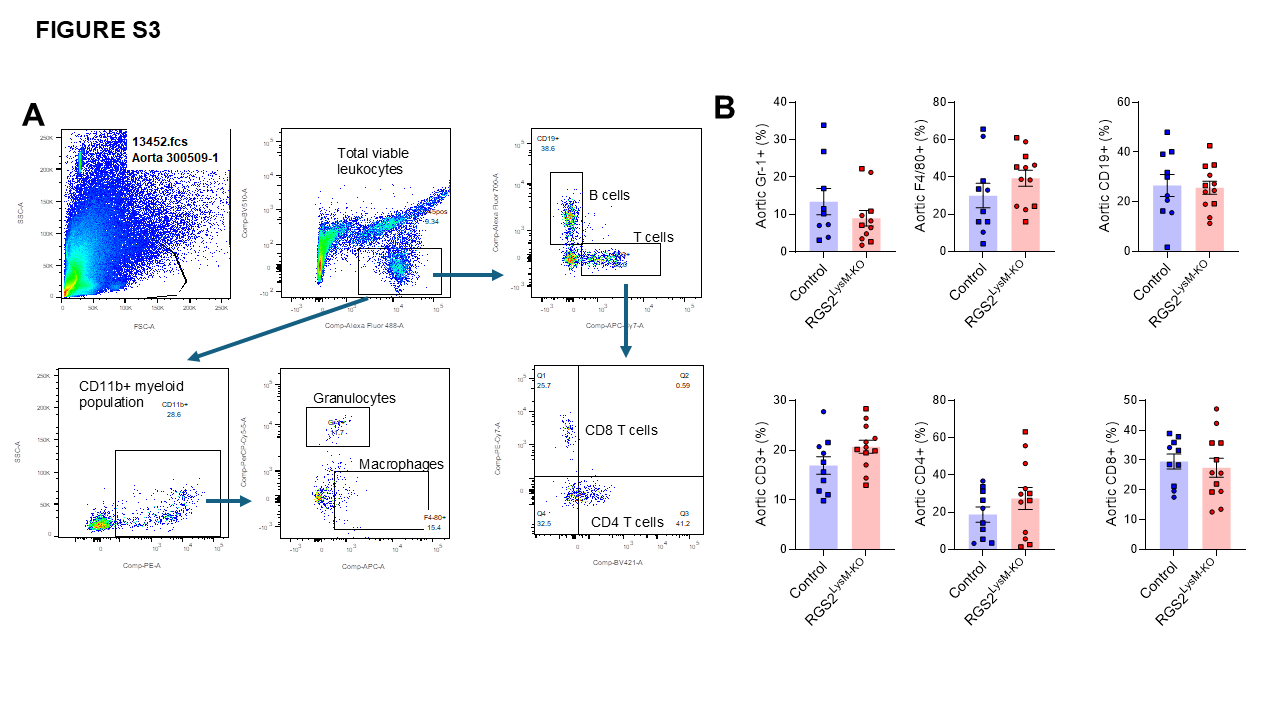


Figure S3: Flow Cytometric Gating and Percentages of Thoracic Aorta Leukocyte Subsets. A) Representative flow cytometry gating strategy for thoracic aorta leukocyte subpopulations from Ang II-treated male mice. B) Percentage of parent gates for Gr‑1⁺ granulocytes, F4/80⁺ macrophages, CD19⁺ B cells (top row), CD3⁺ total T cells, and CD4⁺ helper or CD8⁺ cytotoxic T cell infiltrates (bottom row).


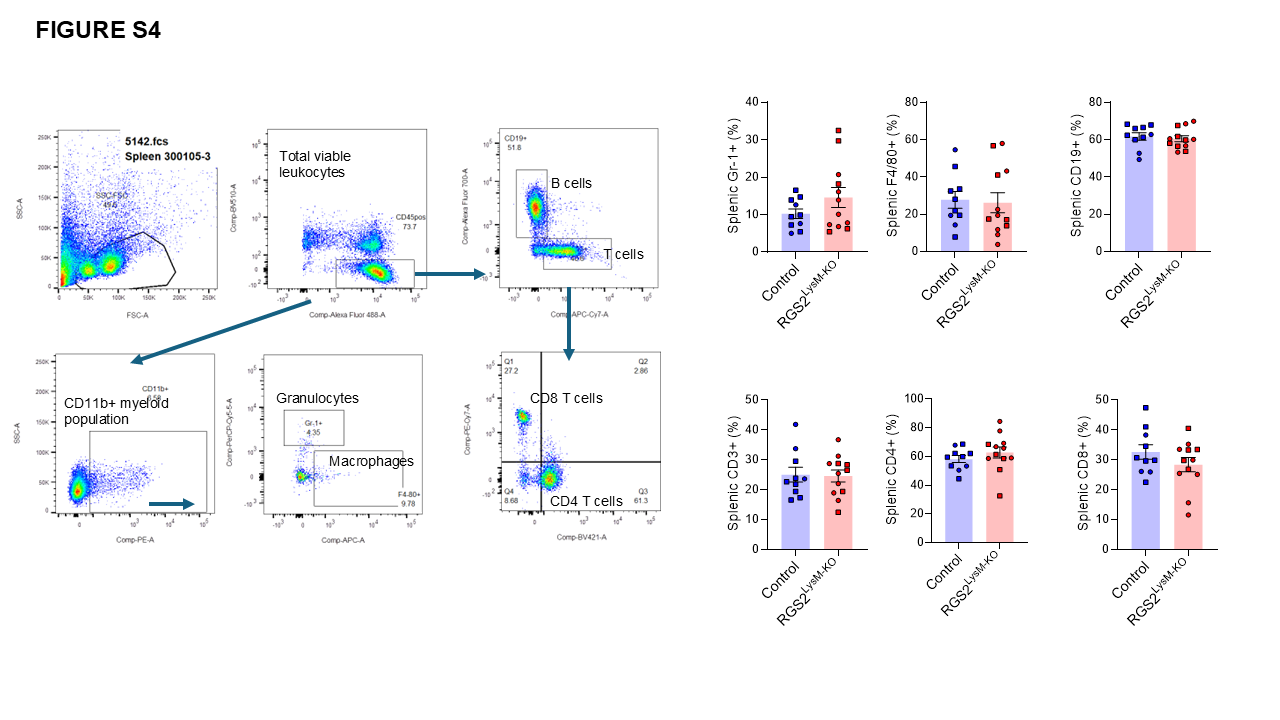


Figure S4: Flow Cytometric Gating and Percentages of Splenic Leukocyte Subsets. A) Representative flow cytometry gating strategy for splenic leukocyte subpopulations from Ang II-treated male mice. B) Percentage of parent gates for Gr‑1⁺ granulocytes, F4/80⁺ macrophages, CD19⁺ B cells (top row), CD3⁺ total T cells, and CD4⁺ helper or CD8⁺ cytotoxic T cell infiltrates (bottom row).
